# Left Ventricular Volumes, Mass and Function normalized to the body surface area, age and gender from CMR in a large cohort of well-treated Thalassemia Major patients without myocardial iron overload

**DOI:** 10.1186/1532-429X-13-S1-P305

**Published:** 2011-02-02

**Authors:** Antonella Meloni, Maria Chiara Dell'Amico, Brunella Favilli, Giovanni D Aquaro, Pierluigi Festa, Elisabetta Chiodi, Stefania Renne, Maria Concetta Galati, Leonardo Sardella, Petra Keilberg, Vincenzo Positano, Massimo Lombardi, Alessia Pepe

**Affiliations:** 1 “G. Monasterio” Foundation and Institute of Clinical Physiology, CNR, Pisa, Italy; 2 Arcispedale “S. Anna”, Ferrara, Italy; 3 P.O. “Giovanni Paolo II”, Lamezia Terme, Italy; 4 A.O. "Pugliese-Ciaccio", Catanzaro, Italy; 5 A.S.L. BA - Osp. “Di Venere”, Bari, Italy

## Introduction

Cardiovascular Magnetic Resonance (CMR) allows an accurate and reproducible quantification of left ventricular (LV) parameters. In Thalassemia major (TM) patients have been reported different “normal” LV values due to chronic anemia and eventually pre-existing iron burdens. Moreover, in this population it is unknown the influence of sex and age on LV parameters and no ranges of normal have been reported using MASS® software. The aim of this study was to establish the ranges for normal LV volumes, mass and ejection fraction normalized to the influence of body surface area(BSA), age and sex from CMR in a large cohort of well-treated TM patients without myocardial iron overload.

## Materials

Among the 923 TM patients who underwent CMR within the MIOT network for the assessment of cardiac iron overload, function and fibrosis, we selected 142 patients with no known risk factors or history of cardiac disease, normal electrocardiogram, no myocardial fibrosis and no myocardial iron overload (all the cardiac segments with a normal T2* value). All patients had been regularly transfused and chelated since early childhood. Moreover, we studied 71 healthy subjects matched for age and sex. LV function parameters were quantitatively evaluated in a standard way by SSFP cine images using MASS® software. LV end-diastolic volume, end-systolic volume, stroke volume, and mass were normalized to BSA (EDVI, ESVI, SVI, massI).

## Results

Table 1 shows the comparison of the CMR parameters with differentiation for sex and age in TM patients and healthy subjects and the cut-off of normality defined as mean - 2 standard deviation (SD). TM patients showed significantly lower BSA than the controls (P<0.0001). Significantly higher EDVI and SVI were found only for males 30 years. Significantly higher LVEF were found only for males < 14 years. In TM patients all LV volumes indexes were significantly larger in males than in females (P<0.0001 in all cases). The EF was not different between the sexes. In males the ESVI and the EF were significant different among the age groups (P=0.006 and P=0.001, respectively). In females no significant differences were detected among the age groups.

**Table 1:** 

	< 14			14-20			20-30			30-40			>=40		
	**TM**	**H**	**P**	**TM**	**H**	**P**	**TM**	**H**	**P**	**TM**	**H**	**P**	**TM**	**H**	**P**

**Males**	*N=7*	*N=7*		*N=6*	*N=6*		*N=25*	*N1=15*		*N=23*	*N=11*		*N=6*	*N=5*	

EDVI (ml/m^2^)	94 ± 18 (58)	75 ± 11	0.034	96 ± 20 (56)	91 ± 20	0.715	103 ± 17 (69)	101 ± 13	0.686	92 ± 15 (62)	80 ± 11	0.222	94 ± 9 (76)	75 ± 11	0.013
ESVI (ml/m2)	31 ± 6 (19)	24 ± 6	0.058	36 ± 8 (22)	35 ± 14	0.760	38 ± 8 (22)	39 ± 9	0.676	32 ± 6 (20)	29 ± 6	0.160	29 ± 6 (17)	28 ± 10	0.816
SVI (ml/m^2^)	63 ± 14 (35)	51 ± 7	0.05	57 ± 12 (33)	56 ± 8	0.868	65 ± 10 (45)	62 ± 9	0.433	59 ± 10 (39)	51 ± 10	0.027	64 ± 8 (48)	47 ± 8	0.007
Mass I (g/m^2^)	57 ± 7 (43)	68 ± 5	0.007	57 ± 13 (31)	71 ± 7	0.043	66 ± 12 (42)	77 ± 12	0.006	62 ± 12 (38)	66 ± 10	0.184	69 ± 16 (36)	74 ± 11	0.536
EF (%)	66 ± 4 (58)	54 ± 6	<0.0001	60 ± 2 (56)	66 ± 14	0.322	63 ± 3 (57)	62 ± 6	0.520	65 ± 3 (59)	65 ± 8	0.972	68 ± 6 (56)	62 ± 9	0.234

**Females**	*N=2*	*N=2*		*N=8*	*N=6*		*N=24*	*N=6*		*N=33*	*N=8*		*N=8*	*N=5*	

EDVI (ml/m^2^)	53 ± 8 (47)	62 ± 4	0.951	81 ± 8 (65)	80 ± 9	0.866	83 ± 16 (51)	78 ± 9	0.499	77 ± 11 (55)	79 ± 10	0.669	82 ± 19 (44)	77 ± 16	0.614
ESVI (ml/m2)	23 ± 1 (21)	22 ±8	0.823	30 ± 6 (18)	31 ± 3	0.789	30 ± 8 (14)	30 ± 4	0.883	26 ± 6 (14)	29 ± 7	0.216	26 ± 8 (12)	28 ± 12	0.974
SVI (ml/m^2^)	40 ± 7 (26)	41 ± 4	0.919	49 ± 3 (43)	49 ± 6	0.892	53 ± 9 (35)	48 ± 6	0.234	51 ± 6 (39)	50 ± 8	0.915	54 ±11 (32)	49 ± 6	0.394
Mass I (g/m^2^)	34 ± 3 (28)	59 ± 19	0.296	47 ± 8 (31)	56 ± 6	0.040	53 ± 9 (35)	54 ± 12	0.893	52 ± 9 (34)	55 ± 13	0.465	51 ± 12 (27)	56 ± 5	0.344
EF (%)	62 ± 3 (57)	46 ± 17	0.388	63 ± 4 (55)	62 ± 4	0.694	65 ± 5 (55)	62 ± 3	0.137	66 ± 5 (56)	63 ± 7	0.271	66 ± 4 (58)	63 ± 7	0.254

## Conclusion

In a large cohort of well-treated TM patients significant differences in LV parameters compared to controls were limited to males < 14 years and > 30 years. Appropriate “normal” reference ranges normalized to BSA, sex and age should be used to avoid misdiagnosis of cardiomiopathy in TM patients.

